# Memory Recall After “Learning by Doing” and “Learning by Viewing”: Boundary Conditions of an Enactment Benefit

**DOI:** 10.3389/fpsyg.2015.01907

**Published:** 2015-12-17

**Authors:** Melanie C. Steffens, Rul von Stülpnagel, Janette C. Schult

**Affiliations:** ^1^Psychology, Social and Economic Psychology, University of Koblenz-LandauLandau, Germany; ^2^Abteilung Kognitionswissenschaft, University of FreiburgFreiburg, Germany; ^3^Psychology, Friedrich Schiller University JenaJena, Germany

**Keywords:** enactment effect, observation learning, free recall, study design, review, SPT, EPT, action sequence

## Abstract

According to common sense, things one has done are remembered better than things done by others that one has observed. On first sight, findings concerning memory for actions appear in line with that preconception: Performed actions (“subject-performed tasks”) appear to be remembered particularly well, and better than observed actions (“experimenter-performed tasks”). A closer look, however, reveals important exceptions regarding this enactment effect. The aim of the present paper is critically evaluating the literature that compares memory for performed and observed tasks. In recognition memory, an enactment effect has regularly been observed. In free recall, however, findings depended on the experimental design: When performed and observed actions were intermixed, an enactment effect was typically found. In contrast, in designs where actions were either all performed or all observed, this was rarely the case. We discuss underlying memory processes, potential moderator variables, open questions, and implications.

*I hear and I forget*.

*I see and I remember*.

*I do and I understand*.

Confucius, ca. 500 b.c.

The aphorism by Confucius, though quite dated, captures what is still widely believed on how to best learn and memorize novel actions and activities: “Learning by doing” appears superior to “learning by viewing” (and verbal learning only appears worst). This assumption is applied to a wide variety of contexts, from instructional design to navigation (e.g., von Stülpnagel and Steffens, 2012, 2013). For example, a frequently provided illustration may read: “I need to drive to remember a route. I will remember nothing as a passenger.” Similarly, “hands-on” learning in instructional design and multi-media learning is often propagated. This common-sense assumption of superior learning after performing actions (i.e., enactment) as compared to other conditions has been addressed in action memory research since the 1980's and is reflected in the axiomatically named “enactment effect.” There is much empirical evidence illustrating that as a rule enactment encoding indeed leads to better memory for simple actions as compared to verbal learning (see Engelkamp, 1998). The gist of research on action memory has been summarized by the statement: “the basic finding in this field is that recall of enacted action phrases is superior to recall of action phrases without enactment” (Engelkamp and Cohen, 1991, p.175). As implied, enactment encoding may also lead to superior memory as compared to observation learning, that is, learning by observing someone else enact (e.g., Golly-Häring and Engelkamp, 2003; Hornstein and Mulligan, 2004). Arguably, the citation from 1991, even if much younger than that by Confucius above, is also somewhat dated. To illustrate our proposal that researchers in the field are still positive that an enactment effect should emerge if experiments are not methodologically flawed, we compiled Table 1. We present anonymous reviewers' comments as reactions to recent reports of experiments from our lab that did not yield better recall after enactment than observation. Notably, it was not our main aim to publish a null effect, but to extend research to novel types of materials, namely action sequences (see Schult et al., 2014). Next to many very helpful comments that improved the presentation of our research, reviewers were much concerned about an action memory report that does not find an enactment effect. As the first three comments in Table 1 illustrate, the postulate of an enactment effect has thus assumed somewhat axiomatic proportions. Importantly, the comments illustrate the suggestion that research practices should be adapted until one finds what one believed to be true in the first place (see Comments 2–3). In conjunction with the widely accepted superiority of enactment encoding, presumably this has led to an emphasis on experimental conditions where enactment encoding leads to superior memory than observation encoding. This is reflected in the solutions shown further below in Table 1 (Comments 4–6) that were suggested by the reviewers in order to avoid or sidestep the “problematic” null effect. To put it bluntly: In order to demonstrate an enactment effect, suggestions made by some reviewers included dropping (or at least altering) our initial research question, or massively gearing our methodology and stimuli toward an enactment effect. Only after forcing some kind of enactment effect by all means, we would be qualified to discuss its limitations. However, this approach may put the cart before the horse.

**Table 1:** **Anonymous reviewers' comments regarding experiments that yielded similar free recall performance in an enactment and in an observation condition**.

1.	“It seems odd to me that in a three-experiment report on the enactment effect there is not a single enactment effect demonstrated […].”
2.	“When the authors failed to get the basic [enactment] effect, they should have gone fully after it […].”
3.	“From my perspective it's critical to establish that the materials, as constructed, are sensitive enough to elicit any kind of enactment effect […].”
4.	“[…] the authors should have placed their initial hypothesis on hold and have gone after the null enactment effect […].”
5.	“If recognition test is more sensitive to pick up the differences, then I suggest they [i.e., the authors] exploit that test to a greater extent, rather than rely on the null effects in free recall […].”
6.	“ I'm particularly sensitive to this issue because I, too, have been in the position of developing my own novel enactment stimuli, which, at first, weren't yielding a significant effect. I had to tinker with them until they did, and only then was it appropriate that I explore more specific questions with my materials.”

Taken together, there is a widespread belief both in society in general and in the scientific community that “learning by doing” is inherently advantageous compared to all other study conditions including “learning by viewing.” However, as we review below, findings concerning the comparison of enactment and observation encoding are rather ambiguous, and whether an enactment effect is observed or not depends on factors such as study design and memory task. After conducting more than a dozen experiments with different stimuli and study conceptualizations, we are confident to claim that a memory advantage of enactment encoding over observation encoding does not generally exist (i.e., independent of study and testing conditions). An enactment effect over observation may emerge, for example, if recognition tasks are used (rather than recall tasks), or by using a within-list (rather than a pure-list) manipulation of study conditions, as we will elaborate below. However, we want to take up the cudgel on behalf of the argument that recall differences between enactment encoding and observation encoding represent—contrary to what is frequently assumed—the exception rather than the rule. The review at hand provides a summary of respective investigations and a theoretical perspective that tries to explain (most of) the existing findings, in addition to suggesting new studies.

## Memory for actions

The paradigm typically used in action memory research involves presenting simple action phrases such as “comb your hair” for a later memory test. In the enactment condition, participants perform an appropriate action for each phrase themselves. A verbal learning condition has most frequently been used as a control condition (for a comprehensive review, see Engelkamp, 1998), but this has several disadvantages. First, left to their own devices, it is an open question which mnemonic strategies, if any, participants use. Second, given more naturalistic action sequences (e.g., to fold a paper frog), it is difficult to capture verbally what exactly should be done in each action step. Most importantly, an observation learning condition appears the closest and therefore most adequate control condition: Everything is held constant but motor performance. For example, one participant (or alternatively the experimenter) may perform the actions, while another participant observes him or her. This allows researchers to pin down the mnemonic consequences of enactment in the absence of any confounding factors.

By performing actions participants are forced to process task-relevant features of action phrases. In line with this idea, it has been found that enactment improves item-specific processing of a phrase's verb and object as well as the verb-object relation (e.g., Knopf, 1995; Kormi-Nouri, 1995; Engelkamp, 1998; Steffens, 1999; von Essen, 2005; Steffens et al., 2006, 2009). It appears that people in the enactment condition focus on the details of what they are doing, and this improves memory for what they have done (i.e., the action verb) with which object (i.e., the action object); this is referred to as item-specific processing.

### Recognition

During recognition, participants are given the verbs, the objects, or the action phrases they have learned, among distractors, and are asked to indicate which ones were presented during study. Recognition tests are particularly sensitive to item-specific information. Indeed, when using these tests, a clear-cut enactment effect compared to observation has been reported (Engelkamp and Krumnacker, 1980; Koriat et al., 1991; Engelkamp and Dehn, 2000; Golly-Häring and Engelkamp, 2003; Mulligan and Hornstein, 2003; Hornstein and Mulligan, 2004; Manzi and Nigro, 2008). In other words, the recognition of phrases such as “light the match,” as well as the recognition of the object (“match”) and the verb (“light”), is improved if they have been enacted rather than observed during study^1^.

### Free recall

During free recall, participants are asked to list as many of the action phrases they have learned before as possible, either on a blank sheet of paper, or verbally, or they are asked to enact them (i.e., performance-based recall). Findings regarding the enactment effect are less clear for free recall. Arguably, being able to recall actions is often more important than only recognizing them when presented. After all, one aim of learning actions is to be able to carry them out at a later point in time (i.e., performance-based free recall). Free recall has been considered to be a function of relational processing in addition to item-specific processing (e.g., Hunt and Einstein, 1981; Hunt and McDaniel, 1993; McDaniel and Bugg, 2008). Relational processing is defined as processing the relations among to-be-learned action phrases, thus providing retrieval routes that can be used for generating the next action if one action has been remembered. For example, relational information can be based on similarities between actions or on the order in which they were presented. During free recall, better processing of item-specific information may compensate poorer processing of relational information, and vice versa, and this trade-off may result in comparable net recall across different study conditions. Although enactment improves item-specific processing, it does not generally enhance relational processing. Particularly, enactment does not enhance relational processing if relations among action phrases do not become salient during enactment (Steffens, 1999). For instance, enactment has not been found to improve clustering based on taxonomic classifications (e.g., gardening activities), but it does increase clustering based on movement-based similarities (e.g., twisting; Koriat and Pearlman-Avnion, 2003). These findings indicate that the type of relations that exist among to-be-learned action phrases moderates findings. Enactment may even hinder the processing of order information, so that after enactment, participants are less able to indicate which action was first in a list, which was second, and so forth (e.g., Engelkamp and Dehn, 2000; also see Olofsson, 1996). In contrast, observing someone enact enhances relational processing more than enactment does, which may result in equal free recall for short lists of unrelated actions (Engelkamp and Dehn, 2000). As the latter finding implies, list length needs to be taken into account when evaluating findings.

#### Within-list designs

When predicting free recall performance after enactment vs. observation, the experimental design needs to be taken into account (Engelkamp and Zimmer, 1997; Engelkamp and Dehn, 2000; Golly-Häring and Engelkamp, 2003). Whereas in a pure-list design participants either enact or observe all action phrases of one list, they switch between these encoding tasks in within-list designs, for example, enacting the first action, observing the second, and so on. Within-list designs have been found to elicit a robust enactment effect in free recall (e.g., Zimmer and Engelkamp, 1984; Dick and Kean, 1989; Engelkamp and Zimmer, 1997; Engelkamp and Dehn, 2000; Golly-Häring and Engelkamp, 2003; Zalla et al., 2010). One possible explanation for this is the disrupting of relational processing during observation through the intervening actions. If good free recall after observation particularly depends on relational processing, and this relational processing is disturbed by intervening actions, then free recall performance deteriorates for observed actions. A second reason for bad recall performance for observed actions intermixed with enacted actions could be selectively displaced rehearsal (see Slamecka and Katsaiti, 1987): Possibly, actions carried out appear more important than those observed, so that relatively more attention is devoted to actions when interspersed with observed phrases (for evidence related to importance, see Schult and Steffens, 2011, 2013).

### Free recall after enactment vs. observation in pure-list designs

A pure-list design in which participants learn all action phrases either by enactment or by observation may be a purer test to investigate the contributions of item-specific and relational information to free recall: In within-list designs, participants need to switch between tasks, and task-switching provokes cognitive costs (e.g., Meiran et al., 2000). Also, a signal is needed which task to carry out (“enact,” “observe”) that may interfere with memory encoding. Arguably, learning something either by enactment or by observation is also highly relevant in applied contexts. For example, one may give someone else detailed instructions on how to make pancakes (enactment: “now crack an egg and add it”). Alternatively, one may watch a video instruction on how to accomplish a task (observation: “now I crack an egg and add it”). We are not implying that taking turns is not relevant in applied contexts (e.g., someone may enact the easier action steps and observe the more complicated ones); instead, we believe that learning either by enactment or by observation is also frequent.

Findings in pure-list designs are mixed. Table 2 summarizes the total of 36 experiments that contrasted free recall of healthy adults after enactment and observation in pure-list designs^2^. As can be seen, superior recall after enactment than observation is rather the exception than the rule. Only seven experiments reported an enactment effect over observation. As indicated above, internal list structure and list length need to be considered when evaluating findings. In Table 2 we additionally included information about object presentation during the study phase and about study-test cycles as further possible moderating variables. Two experiments that manipulated object presentation (object present vs. imagined) within a list showed comparable recall improvement after object presentation by enactment and observation (Engelkamp and Zimmer, 1983, 1997). Thus, whether participants use real objects during the study phase or enact phrases symbolically, pretending to use an object, appears not to affect recall performance differentially for both encoding tasks. Similarly, comparable effects have been obtained whether verbal or performance-based free recall is used (i.e., reporting actions verbally during the memory test or performing actions again, e.g., Steffens, 2007).

**Table 2:** **Experiments comparing free recall after Enactment (***E***) and Observation (***O***) in pure-list designs**.

**Number**	**Source**	**Exp. Number**	**Direction and size of effect (*d*)**	**List length**	**Internal list structure**	**Objects during study phase**	**No. of study-test cycles**
1	Cohen, 1981	1	*E* = O	15	Unrelated	Real	2
2	Cohen, 1983	1	*E* = O	21	Unrelated	Real	2
3	Cohen and Bean, 1983	-	*E* = O	12	Unrelated	Real	5
4	Cohen et al., 1987	1	*E* = O	24	Unrelated	Real	1
5		2	*E* = O	20	Unrelated	Real	1
6		4	*E* = O	18	Unrelated	Real	1
7		5	*E* = O	23	Unrelated	Real	1
8	Engelkamp and Dehn, 2000	1	*E* = O	8	Unrelated	None	8
9		2	*E* = O	8	Unrelated	None	8
10		4	***E*** > **O**	24	Unrelated	None	4
11		5	*E* = O	24	Unrelated	None	4
12	Engelkamp et al., 2003	1	*E* = O	8	Unrelated	None	8
13		2	*E* = O	8	Unrelated	None	8
14		3	*E* = O	8	Unrelated	None	8
15	Engelkamp and Krumnacker, 1980	–	*E* = O	48	Unrelated	None	2^a^
16	Engelkamp and Zimmer, 1983	–	***E*** > **O (1.19)**	48	Unrelated	Real vs. none	1
17	Engelkamp and Zimmer, 1997	2	*E* = O	18	Unrelated	Real vs. none	1
18		3	***E*** > **O (1.01)**	48	Unrelated	Real vs. none	1
19	Golly-Häring and Engelkamp, 2003	1	***E*** > **O (0.83)**	24	Object-category	None	4
20		2	***E*** > **O**	12	Object-category	None	6
21		3	***E*** > **O**	8	Object-category	None	8
22		4	***E*** > **O (1.02)**	6	Object-category	None	8
23	Nadar and McDowd, 2008	–	*E* = O	20	Unrelated	Real	1^b^
24	Schult et al., 2014	1a	*E* = O (u)	24	Unrelated vs. sequence	None	4
			*E < O (0.34, s)*				
25		2	*E* = O	60	Unrelated vs. sequence	None	1
26	Steffens, 2007	1	*E* = O	25	Sequence	Real	1
27		2	*E* = O	68	Sequence	Real	1
28	von Stülpnagel et al., 2015b	1	*E* = O	25, 14	Sequence	Real	2^c^
29		2	*E* = O	25, 14	Sequence	Real	2
30		3	*E* = O	25, 14	Sequence	Real	2
31		4	*E* = O	25, 14	Sequence	Real	2
32	Online Appendix	5	*E < O (0.38)*^d^	25, 14	Sequence	Real	2
33	von Stülpnagel et al., in press	1	*E* = O^e^	9–30	Sequence	Real	5
34		2	*E* = O^f^	9–30	Sequence	Real	5
**UNPUBLISHED EXPERIMENTS**							
35	Schult et al., 2015a	1	*E* = O	60	Unrelated	None	1
36	von Stülpnagel et al., 2015a	1	*E* = O	48	Unrelated vs. sequence	None	1
37	Schult et al., 2015b	1	*E* = O	46, 47	Sequence	Real	2
38		2	*E* = O	46, 47	Sequence	Real	2

a *Recall was assessed after a study, a recognition, and again a study phase*.

b *Each participant took part in different experimental conditions (within subject), but there was only one cycle for each task (e.g., enactment)*.

c *Given there was a practice cycle, one could also argue there were 3 cycles, but only 2 were evaluated*.

d *There was an interaction: A negative enactment effect was obtained for one of the sequences (i.e., one study-test cycle)*.

e *An enactment effect was found for the longest of the five sequences (assembling a Lego model) that was not replicated in the subsequent experiment*.

f *Pictorial learning outperformed both enactment and observation for one of the five sequences (creating a computer graph), which was totally unexpected and not found in Experiment 1*. *Statistically significant enactment effects are printed in bold*.

#### Number of study-test cycles

Five experiments that used four or more study-test trials reported an enactment effect aggregated across trials. Each study phase was followed by a recall test, another study-test cycle, and so on. One may assume that enactment of single actions is an unusual encoding task (McDaniel and Bugg, 2008)—people do not routinely perform lists of unrelated actions (let alone pantomimically). It is thus possible that an enactment effect emerges in later test trials when participants have become familiar with enactment-task requirements. It appears that the available evidence provides some support for this idea (but see below).

#### List structure

Another potential moderator is the internal list structure; in other words, whether relations among list elements are given. For example, the objects of several action phrases could be related to each other (e.g., animals, fruit, etc.). Alternatively, there could be no internal list structure (i.e., unrelated lists). We first turn to the evidence on the list-length hypothesis for unrelated lists and then look at the two kinds of internal list structure that have been investigated: object relations and action sequences.

#### List length

Only three out of 23 experiments presenting unrelated lists demonstrated better free recall after enactment than observation. These three experiments used rather long lists containing 24 or 48 action phrases. Indeed, list length has been discussed as a factor moderating the enactment effect (Engelkamp, 1998; Engelkamp and Dehn, 2000). Those authors argued that in lists in which no categorical-relational information is offered, participants in the observation condition spontaneously use serial order information at recall, whereas participants in the enactment condition use item-specific information. Better processing of order-relational information after observation may compensate for poorer item-specific processing, leading to comparable net recall; however, as was argued further, with increasing list length, order information becomes less efficient as a retrieval route. Consequently, an enactment effect over observation was expected if a sufficiently long list of (20 or more) unrelated actions was learned (Golly-Häring and Engelkamp, 2003). In line with a list-length hypothesis for unrelated lists all nine observation-enactment comparisons in Table 2 that used *short lists* of fewer than 20 actions reported comparable free recall performance. Fourteen experiments used *long lists* with 20 or more actions. Contradicting the list-length hypothesis, 11 of them reported no enactment effect. A closer inspection of Table 2 suggests that an enactment effect as compared to observation could be more likely for *very long lists*. Several of the comparisons using lists of 48 actions reported the enactment effect; yet only one out of seven experiments using medium list lengths of 20–24 actions did. Thus, enactment could be advantageous for recalling very long lists of action phrases if no salient relations between action phrases are provided. However, given the number of experiments using such long lists that did *not* yield an enactment effect, the list-length hypothesis should be regarded with caution. It appears safe to conclude that for lists with up to 24 unrelated actions enactment and observation are similarly effective encoding conditions.

#### Object-related actions

In contrast to the small number of enactment effects observed in lists of unrelated actions, all four experiments that presented lists of actions related by object categories demonstrated an enactment effect. Golly-Häring and Engelkamp (2003) presented lists based on the categories of objects involved in the action (e.g., tools or animals). The authors assumed that relational information based on object similarities is equally available after enactment and observation because categorical-relational information is well-established in long-term memory and spontaneously activated when object exemplars are encountered. To the degree that categorical information is efficiently processed in both encoding tasks, the superior item-specific memory after enactment should lead to an enactment effect in free recall. In line with this reasoning, those authors found an enactment effect across four experiments in free recall when exemplars of different categories were presented in a random order or when all actions belonged to the same category. They also reported comparable clustering according to object category for both encoding tasks.

#### Action sequences

However, it should be noted that only a small number of experiments compared enactment and observation using lists of related actions, and relations were restricted to object similarities. An entirely different type of relational information is that present in action sequences such as “making clay” or “building a bird feeder” (e.g., Steffens, 2007). Arguably, learning such a sequence is more common in everyday life than learning unrelated or object-related lists of actions. Moreover, relational information is more diverse and thus less obvious in action sequences than in artificial lists of actions as described above that comprised only relations well-established in long-term memory. In order to learn a new sequence of action steps that are basically known, a sequence of actions needs to be remembered that comprises different (typically unrelated) objects, some of them repeated; it comprises “in-order-to relations” and “enable relations” (Lichtenstein and Brewer, 1980); it comprises steps that may appear totally unrelated unless in this specific sequence that is to be learned. For example, unless you repair a bike's flat tire, “tube,” and “water” may be quite unrelated objects; and in order to make pancakes, some steps have to be finished before others can be begun, whereas other orders are loose. Possibly, you use silverware several times when making pancakes, with different actions (i.e., verbs). Therefore, given this variety of relations that need to be processed, it is possible that learning such sequences of actions does not profit from enactment as compared to observation as much as learning simplified lists based on transparent object categories. Indeed, as Table 2 shows, none of the 14 experiments that compared action sequence learning by enactment and observation found an overall enactment effect in free recall (11 of them are published, three are unpublished experiments from our lab). Two out of the 14 experiments instead yielded better free recall after observation than enactment. In other words, people who had observed someone perform a new sequence of actions were better able to reproduce that sequence of steps than those who had performed the task themselves. Whereas an enactment effect was found for one out of five sequences in one of the 14 experiments, this finding was not replicated in the follow-up experiment that used the same materials. In that follow-up experiment, instead, pictorial learning, involving neither performing nor observing actions, was superior to enactment and observation for another of the five sequences (von Stülpnagel et al., in press). During pictorial learning, participants saw the same illustrated stepwise instructions as did participants in the enactment condition. The evidence thus appears quite clear that learning novel sequences of actions in order to perform them later does not profit from enactment compared to observation.

### Relational processing in action sequences

As reviewed above, free recall is assumed to be based on both item-specific and relational information. Thus, similar performance after enactment and observation may result from a trade-off: superior item-specific processing in the enactment condition (see recognition findings), but superior processing of novel relations in the observation condition (e.g., Engelkamp and Dehn, 2000). For action sequences, there is some evidence of superior item-specific processing in the enactment than observation condition (e.g., an enactment effect in recognition memory: Schult et al., 2014, Experiment 1b). Some evidence of superior relational processing in the observation as compared to the enactment condition has also been published (Steffens, 2007; Schult et al., 2014). However, in addition to those four published experiments from our lab, four additional, unpublished experiments, using materials and procedures very similar to the published ones, did not yield any hints that relational processing is better after observation than enactment (e.g., ordered recall, clustering, and input-output correspondence after both encoding conditions were comparable). At the same time, those experiments also yielded comparable free recall after observation and enactment. Two of those experiments (Schult et al., 2015a; von Stülpnagel et al., 2015a) were from the same set of experiments as the published ones (Schult et al., 2014), but had been suggested to be cut from the paper by reviewers because of the null findings. Two additional experiments tested whether participants in the observation condition understand more quickly than those in the enactment condition what the topic of the action sequence is (Schult et al., 2015b; see Discussion section for details). Memory organization was comparable in both encoding conditions. Taken together, we conclude that the evidence is mixed concerning the question whether observation leads to better organization of action sequences than enactment, or whether some as yet unknown factor contributes to the comparably good recall performance after observation.

## Discussion

Enactment as compared to observation improves recognizing actions. As a rule, enactment also leads to superior free recall when compared to observation if enacted and observed actions are intermixed. However, in a pure-list design (i.e., some participants enact all actions, others observe all actions), enactment and observation have yielded similar memory performance in the majority of the experiments that we are aware of. An enactment effect appears somewhat more likely first, if there are several study-test cycles and second, if very long lists of unrelated actions have to be recalled. Third, if salient relations among action phrases are given, based on objects, an enactment effect is more likely to emerge. However, this is not the case if the actions are embedded in sequences such as “folding a paper frog” or “tying a knot,” where no single experiment has yielded better verbal free recall or performance-based free recall after enactment than observation. Arguably, findings for such sequences have more important implications for everyday learning than findings with artificial lists based on object similarity.

How can we explain this pattern of findings? In line with the theoretical introduction of this paper, enactment appears to improve item-specific processing of each action phrase: People appear to focus on the details of what they are doing, and this improves recognition memory for each single action, on average. As compared to recognition, free recall is a more difficult task, requiring retrieval routes. Whereas there is some evidence that spontaneous “pop-out into memory” is improved by enactment (Zimmer et al., 2000), there is little evidence that retrieval is generally better after enactment than observation. On the contrary, observation appears to often enhance memory organization of action sequences as compared to enactment. Note, however, that the evidence is inconsistent if unpublished experiments on action sequences are taken into account.

If participants in an observation condition focus less on the details of each action than those in the enactment condition, they could become aware of the goals of a sequence of actions earlier (i.e., of the “big picture”). In two experiments, we specifically tested this idea (Schult et al., 2015b). For example, all participants went through the steps of crafting a ghost puppet, either performing the steps or observing them. Several times they were interrupted, and we used explicit as well as implicit tests of their awareness (e.g., a word completion task including word fragments such as “gh”). No evidence was obtained that participants in the observation condition understood more quickly what the task was about than those in the enactment condition. Whereas a null finding is always ambiguous, this indicates at least that more sensitive tests are needed to find differences in the awareness of action sequence goals between enactment and observation. Taken together, some, but not all evidence supports the view that observation leads to superior memory organization than enactment.

One theoretical reason why an enactment effect may emerge is that enactment encoding provides additional memory markers as compared to observation and verbal encoding (Engelkamp, 1998). In spite of the fact that several studies presented convincing evidence that motor information is not the source of the enactment effect (e.g., Helstrup, 2005), it could be argued that the multi-modal encoding during enactment establishes deeper memory traces, which benefits long-term retrieval performance. The paradigm applied in action memory research normally evaluates memory performance within minutes after the study phase^3^. Testing the effects of longer retrieval intervals on memory performance after enactment and observation, especially regarding item-specific and relational encoding strategies, appears to be a worthwhile research topic. However, it is also possible that such deep memory traces are automatically established during observation as well (e.g., Rizzolatti, 2005; Iacoboni, 2005). For example, an experiment on directed forgetting demonstrated that neither young nor old participants were able to intentionally forget actions that had been carried out, as compared to those learned verbally (Earles and Kersten, 2002). This finding supports the idea that enactment encoding provides additional memory markers. But as no observation condition was included in that study, it is an open question whether observation would have left comparably robust memory traces.

Also, it is surprising that experiments comparing cued recall after enactment and observation are scarce (for an exception, see Feyereisen, 2009). Such research should be done to test whether the integration between the verb and object of an action phrase profits from observation as much as from enactment. However, it should be noted that given performance-based recall of action sequences, the distinction between free recall and cued recall is blurred: If objects are present that need to be used during action execution (e.g., “now, make pancakes again”), one could argue that the test is an object-based cued recall of the action sequence, rather than a free recall. The respective studies found similar performance after an enactment and an observation condition. Thus, it is likely that cued recall is similar after enactment and observation learning.

Studies on action memory frequently impose strict time limits for studying and retrieval. A recent study suggests that recall differences between enactment encoding and verbal encoding increase with expanding recall time (Spranger et al., 2008; also see Kubik et al., 2014a). Again, an observation condition was not included and should be in future research. In other words, we cannot exclude the possibility that enactment would yield better recall than observation if participants were given more time (than a few minutes) to retrieve all the actions they remembered.

What else differs between the enactment and the observation condition? Enactment is an unfamiliar task (McDaniel and Bugg, 2008) that may draw attention away from memorizing retrieval routes. At the same time, being able to perform all the required actions may suggest to participants that they could easily do it again—after all, they have already succeeded once. For instance, it was recently demonstrated that participants during enactment encoding, as compared to observation and other study conditions, thought the memory task was very easy, and they were afterwards surprised about their bad memory performance (von Stülpnagel et al., 2015b). Possibly, if the study and recall phases were extended, the disadvantages of such a novel task would fade, and participants would profit more from good item-specific encoding after enactment. Enactment effects obtained with several study-test cycles could hint into this direction (see Table 2). However, Kubik and colleagues recently demonstrated that enactment effects, as compared to verbal learning, disappeared in later study-test cycles (Kubik et al., 2014a). These findings speak against the idea that participants may profit more from good item-specific encoding after enactment in later study-test cycles.

Another theoretical idea has also missed empirical support up to now. We reasoned that, if observation draws attention to the overall sequence structure, but enactment helps to learn the details (i.e., item-specific processing), then the optimal combination of encoding conditions would be to first observe, then enact the same sequence. Thus, observation would provide the “big picture,” and enactment would help to learn the details. However, as compared to enacting the sequence twice, both enactment-then-observation and observation-then-enactment yielded *worse* recall (Gottschlich, 2013). Observing the sequence twice yielded intermediate recall performance that was not significantly different from performance after enacting the sequence twice. That experiment used the “backpack packing” sequence described by Steffens (2007, Experiment 1). Thus, findings rather suggest that consistently learning a novel action sequence by enactment *or* by observation is superior to switching between study conditions. Possibly, the routine obtained by repeating the same study condition, be it enactment or observation, yields a memory advantage as compared to familiarizing one-self with a different task on the second cycle.

The question remains *why* the belief in the enactment effect is so robust despite contrasting evidence and convincing counter-examples. We can think of two reasons. First, the enactment of novel activities (i.e., “learning by doing”) in everyday life as compared to the lab-based studies discussed in this manuscript is often self-paced. To illustrate: A person building a bird feeder according to a manual can study the instructions until sufficient comprehension is accomplished. Execution can be as thorough as desired. Corrections can be made. If, in contrast, the building of the bird feeder is demonstrated to a group by a skilled instructor, these opportunities to regulate the encoding process and thus one's cognitive load are more limited. Additionally, the overall time span available to encode the action steps is likely to be shorter than during self-paced enactment. In other words, lay people's comparison of “learning by doing” and “learning by viewing” may be frequently confounded by factors such as exposition time and mental effort spent. Unfortunately, these confounds are rather hard to overcome in lab studies: Self-paced enactment encoding of one participant could be yoked to an observing participant to control exposition time. However, it appears likely that the extended observation of a person fiddling around with study materials and correcting mistakes may lose the observer's attention rather quickly. A potential solution could be to provide observing participants with the possibility to study video instructions at their own pace. Nevertheless, differences in self-regulated or other-regulated study time may be one source of the belief in the enactment effect and thus worthwhile of future investigation.

A second reason for the frequent assumption of the superiority of “learning by doing” in everyday life may result from a confound of enactment with generation. The generation of results by cognitive operations has often been shown to benefit memory performance (e.g., Crutcher and Healy, 1989; Steffens and Erdfelder, 1998). Because the typical study materials in action memory research are designed to be as simple, clear, and unambiguous as possible, they hardly require a cognitively demanding generation of the action. In contrast, many everyday activities (i.e., cooking, handicraft work) allow some degrees of freedom when enacted, but not when observed. This could be experimentally accomplished by study materials without a strict sequential order, where all action steps as well as the final state of the activity need to be generated during the study phase (e.g., a participant would receive the general instruction to build a Lego house along with pictures of the finished house). Following this line of thought, it would be a challenging task to separate the cognitive costs of action planning and motor information during the encoding of such action sequences (see Knopf et al., 2005, for such an approach on memory for simple, unrelated actions).

In a nutshell, it appears that the net result—remembering to perform a series of actions when required to do so—is similar after learning by enactment and learning by observing someone else enact. In free recall in pure-list designs, the most likely outcome is a trade-off, with better item-specific processing during enactment and somewhat better organization during observation yielding similar recall.

Practically speaking, it appears that novel sequences consisting of known action steps can be performed as well after observing someone else as after performing them one-self. Even if new motor sequences are acquired, performing is not always superior to observing (Larssen et al., 2012). Only if the aim is recognizing performed actions, enactment leads to better memory than observation. And if some action steps are performed, but others are observed, the observed ones tend to be more easily forgotten. Of course, memory recall after observation should only be as good as memory recall after enactment if all relevant information becomes available in both conditions. For example, if teaching someone a new computer program, the teacher has to make sure that each mouse click and each shortcut used on the keyboard is shown and/or told to the observer. This could be easier to accomplish if the novice operates all devices, instead of the teacher. Similarly, if passengers stop paying attention to the route, it is of little surprise that they do not remember it afterwards. Thus, one final reason why people believe in “learning by doing” could be that information is regularly omitted or unattended in “learning by viewing” or learning by instruction unless one makes an effort to the contrary. If such an effort is made, as in the experiments reviewed here, recall differences are often negligible between an enactment and an observation learning condition—in spite of lay people's and experts' convictions.

## Author contributions

JS and RS compiled the articles reviewed. MS, RS, and JS contributed towards writing the paper.

### Conflict of interest statement

The authors declare that the research was conducted in the absence of any commercial or financial relationships that could be construed as a potential conflict of interest.

## Footnotes

^1^Whereas several studies comparing enactment with verbal learning have used cued-recall tests to investigate verb-object integration (e.g., Bäckman and Nilsson, 1991; Kormi-Nouri, 1995), there is a lack of research comparing cued recall after enactment and observation (for an exception, see Feyereisen, 2009).

^2^We refrained from a statistical meta-analysis of the findings because the necessary information (e.g., *SDs*, exact statistics for each condition) is missing in many cases, particularly in the case of null findings.

^3^Several experiments investigated delayed recall, but did not include an observation condition (Knopf and Neidhardt, 1989; Nilsson et al., 1989; Kubik et al., 2014b).
